# The kinematics and strategies of recovery steps during lateral losses of balance in standing at different perturbation magnitudes in older adults with varying history of falls

**DOI:** 10.1186/s12877-020-01650-4

**Published:** 2020-07-20

**Authors:** Shani Batcir, Guy Shani, Amir Shapiro, Neil Alexander, Itshak Melzer

**Affiliations:** 1 Department of Physical Therapy, Faculty of Health Sciences, Ben-Gurion University of the Negev, Beer-Sheva, Israel; 2 Schwartz Movement Analysis & Rehabilitation Laboratory, Department of Physical Therapy, Recanati School for Community Health Professions, Faculty of Health Sciences, Ben-Gurion University of the Negev, P.O.B. 653, 84105 Beer-Sheva, Israel; 3 Department of Information Systems, Faculty of Engineering Sciences, Ben-Gurion University of the Negev, Beer-Sheva, Israel; 4 Department of Mechanical Engineering, Faculty of Engineering, Ben-Gurion University of the Negev, Beer-Sheva, Israel; 5 Division of Geriatric and Palliative Medicine, Department of Internal Medicine, University of Michigan, Veterans Affairs Ann Arbor Health Care System Geriatrics Research Education and Clinical Center (GRECC), Ann Arbor, MI USA

**Keywords:** Falls, Recurrent fallers, Balance recovery reaction, Single-step threshold, Multiple-step threshold

## Abstract

**Background:**

Step-recovery responses are critical in preventing falls when balance is lost unexpectedly. We investigated the kinematics and strategies of balance recovery in older adults with a varying history of falls.

**Methods:**

In a laboratory study, 51 non-fallers (NFs), 20 one-time fallers (OFs), and 12 recurrent-fallers (RFs) were exposed to random right/left unannounced underfoot perturbations in standing of increasing magnitude. The stepping strategies and kinematics across an increasing magnitude of perturbations and the single- and multiple-step threshold trials, i.e., the lowest perturbation magnitude to evoke single step and multiple steps, respectively, were analyzed. Fall efficacy (FES) and self-reported lower-extremity function were also assessed.

**Results:**

OFs had significantly lower single- and multiple-step threshold levels than NFs; the recovery-step kinematics were similar. Surprisingly, RFs did not differ from NFs in either threshold. The kinematics in the single-step threshold trial in RFs, however, showed a significant delay in step initiation duration, longer step duration, and larger center of mass (CoM) displacement compared with NFs and OFs. In the multiple-step threshold trial, the RFs exhibited larger CoM displacements and longer time to fully recover from balance loss. Interestingly, in the single-stepping trials, 45% of the step-recovery strategies used by RFs were the loaded-leg strategy, about two times more than OFs and NFs (22.5 and 24.2%, respectively). During the multiple-stepping trials, 27.3% of the first-step recovery strategies used by RFs were the loaded-leg strategy about two times more than OFs and NFs (11.9 and 16.4%, respectively), the crossover stepping strategy was the dominated response in all 3 groups (about 50%). In addition, RFs reported a lower low-extremity function compared with NFs, and higher FES in the OFs.

**Conclusions:**

RFs had impaired kinematics during both single-step and multiple-step recovery responses which was associated with greater leg dysfunction. OFs and NFs had similar recovery-step kinematics, but OFs were more likely to step at lower perturbation magnitudes suggesting a more “responsive” over-reactive step response related from their higher fear of falling and not due to impaired balance abilities. These data provide insight into how a varying history of falls might affect balance recovery to a lateral postural perturbation.

**Trial registration:**

This study was registered prospectively on November 9th, 2011 at clinicaltrials.gov (NCT01439451).

## Background

An increased susceptibility to falling sideways is a major cause of hip fracture and functional disability [1–3]. The impaired ability to recover from an unexpected lateral loss of balance is especially relevant to falls among older adults. When balance is lost unexpectedly to the right or left, arm lift [4] and trunk movements [5] are used to decelerate the center of mass (CoM) motion and preserve balance at minor perturbation magnitudes. A lateral recovery step is used at higher perturbation magnitudes, and the foot is placed more to the right or left to preserve balance during locomotion [6–9] and during standing [10–12] to adequately regulate the relationship between the body’s CoM and the base of support (BoS) provided by the feet to prevent a fall. The spatiotemporal characteristics of the recovery steps (e.g., direction, timing, and length) need to match the requirements for controlling balance. Many past studies of the ability to recover from an unexpected loss of balance have focused on postural strategies and kinematics in the anterior–posterior direction, and explored age-related differences. The lateral direction kinematics in older adults with a varying history of falls and the strategies they use to recover from balance loss at increasing perturbation magnitudes has been less investigated.

In the current study, we aimed to investigate the differences in balance recovery to a lateral balance loss between non-fallers (NFs), recurrent fallers (RFs), i.e., high-risk fallers, and older adults who had fallen only once (OFs), which could indicate either a slightly impaired balance control system or an isolated event that does not truly represent their balance ability. We compared the kinematic characteristics of the recovery stepping at the single step- and multiple step-thresholds levels. These step threshold levels were shown previously to be independent predictors of a future fall [13–18]. In addition, we compared the recovery step kinematic and stepping strategies used by NFs, OFs, and RFs in single-step and multiple-step reactions that were observed at increasing magnitudes of perturbations. These investigations may help in understanding mechanisms of balance recovery and future fall risk.

We first hypothesized that compared with NFs, step threshold levels will be reduced in OFs, and even more so in RFs and that RFs will have a higher probability of taking steps to recover their balance (higher probability of stepping reactions). Additionally, we hypothesized that these step threshold levels will be negatively associated with fear of falls and positively associated lower extremity function. Our second hypothesis was that the kinematics of the first recovery step during single-step and multiple-step threshold trials will be impaired in RFs, especially step initiation duration, as well as their ability to control the CoM motion. Thus, the total time to fully recover balance during the single- and multiple-step threshold trials will be delayed compared with OFs and NFs. Third, we hypothesized that RFs will use a different first step recovery strategy, i.e., less crossover strategy compared with NFs and OFs, since crossover stepping requires a more complex swing trajectory to move one foot across the other foot while circumventing the stance leg, and this may result in a delay in step initiation and balance recovery in RFs. Fourth, we hypothesized that the kinematics in single-step and multiple-step reactions along all perturbation magnitudes will be impaired in RFs.

## Methods

### Study population

Eighty-three community-dwelling older adults were recruited using flyers, advertisements, and personal contacts: 51 NFs, 20 OFs (fell once in the past year), and 12 RFs (fell twice or more in the past year). Participants were 70 years or older and independent in daily living activities. They were excluded if they met any of the following criteria: (a) blindness or serious vestibular impairments (Meniere’s disease, dizziness); (b) an inability to ambulate independently; (c) a score < 24 on the Mini-Mental State Examination; (d) symptomatic severe cardiovascular disease; (e) a neurological disorder such as stroke, Parkinson’s, or multiple sclerosis; (f) an orthopedic acute disorder such as total hip or knee replacement; (g) severe rheumatoid arthritis; or (f) cancer (metastatic or under active treatment). Prior to participation, all subjects signed an informed consent form that was approved by the Helsinki Committee of Barzilai University Medical Center in Ashkelon, Israel (ClinicalTrials.gov Registration number, NCT01439451). This analysis is a supplementary study based on the baseline examination of a randomized control trial that were reported earlier [19, 20].

### Study protocol

Participants stood with their feet close together (i.e., heels and toes touching) on a perturbation device that provided multi-directional unannounced underfoot perturbations [21]. They were instructed to “react naturally” to a total of 26 random right and left unannounced surface translations (1–13 perturbation magnitudes levels) that systematically increased from low to high magnitudes. The magnitudes of perturbations (scales 1–13) was expressed in terms of horizontal surface displacement (1–13 cm), displacement time (0.30–0.80 ms), velocity (6–28 cm/ms), and acceleration (25–128 cm/ms^2^) (see Supplementary Table 1). The order of the perturbation magnitude was not randomized since we aimed to identified the participants single-step and multiple-step threshold levels. We found earlier [unpublished] that when older adults are exposed to a perturbation that evoked stepping response they tended to perform stepping even in a very low perturbation magnitude. At any point, they were able to take a rest break or stop the experiment. To prevent injury, participants wore a loose safety harness designed to arrest a fall to the ground and yet allow balance recovery reactions. Examiners were blinded to the fall history of the subjects.

### Descriptive measures

The presence of single- and multiple-step responses and the Single-step and multiple-step threshold levels as well as first step recovery strategies following a loss of balance were identified by a PhD student in physical-therapy using Windows Media Player, allowing image pauses, slow motion, and running of the image backwards and forwards. We also verified an existence of each recovery step by 3D kinematic data. This method showed excellent inter-observer reliability for single-step and multiple-step thresholds (ICC2,1 = 0.978 and ICC2,1 = 0.971, respectively; *p* < 0.001), an excellent total inter-observer kappa score for balance recovery strategies (0.896, *p* < .0.001) and inter-observer percentage agreements of 85–100% for leg strategies among older adults [22]. The single-step threshold level was defined as the minimum perturbation magnitude that consistently elicited a single compensatory step for at least two consecutive perturbation magnitudes. The multiple-step threshold was defined as the minimum perturbation magnitude that consistently elicited a sequence of recovery steps. During the analysis, we explored the step recovery strategies during all single-step and multiple-step trials. We also calculated the probability of stepping, i.e., the number of change-of-BoS trials, i.e., recovery step trials divided by the total number of trials.

Several step strategies following right/left perturbations were identified: 1) LLSS – loaded leg side step, i.e., performing the first step after the perturbation in the opposite direction of the platform translation; 2) ULSS – unloaded leg side step, i.e., performing the first step in the same direction of the platform translation; 3) COS – crossover step; stepping with the unloaded leg in the opposite direction of the platform translation while swinging the leg over the loaded leg; 4) Leg Abduction – abducting the unloaded leg and standing on one leg only; The ULSS, COS, and leg abduction are considered “unloaded step responses” since the “responding foot” is unloaded as a result of lateral platform translation. These strategies allow a more rapid foot lift than during the loaded leg strategy. The leg abduction responses were not considered in the kinematic analysis because of the nature of the response that causes a very long swing times and very short step. In addition, leg collisions (Col) also explored i.e., collision between the swinging leg and loaded leg during COS.

### Kinematic analysis of stepping

During the single- and multiple-step reaction trials, 3D kinematic data were acquired through the Ariel Performance Analysis System (APAS, Ariel Dynamics Inc.; CA, USA). Two video cameras were mounted at a 45° angle between each camera and the subject’s standing position, at a height of 2.5 m and 7 m in front of the perturbation system. The two video cameras simultaneously recorded the motion of eight reflective markers with a sampling frequency of 60 Hz. The markers were placed at the anterior midpoint of the ankle joints, anterior superior iliac spine, acromion processes, and radial styloid processes. Views from both cameras were mapped onto a 3D coordinate system using an internal direct linear transformation algorithm and were digitized, transformed, and smoothed using a low-pass filter (Butterworth second-order forward and backward passes) with a cut-off frequency of 5 Hz. This approach was shown to be valid and reliable, i.e., a mean point estimate error of less than 3.5 mm, 1.4 mm mean linear error, and 0.26° mean angular error [23]. The following events were identified: the surface translation was detected as the first mediolateral deviation of the marker placed on the mechatronic system greater than three standard deviations from the average baseline noise; step initiation was defined at the first mediolateral deviation of the marker placed on the swinging leg ankle joint more than 4 mm from the average baseline after the surface horizontal translation; foot contact on the ground, i.e., the end of the mediolateral deviation of the marker placed on the swinging foot completing the first recovery step.

To help control for the balance challenge to each participant’s ability, we compared the kinematic parameters of stepping after identifying the participants single-step and multiple step-threshold levels. we also compared the kinematic step parameters in the single-step and multiple-step reactions along all perturbation magnitudes. The following kinematic parameters were extracted: 1) The step initiation duration in milliseconds (ms) was calculated as the time from surface horizontal translation to step initiation, 2) First recovery stepping duration in ms was calculated as the time from surface translation to foot-contact on the ground, 3) First compensatory step length was calculated as the Euclidian distance in centimeters (cm) that the ankle markers displaced from step initiation to first step recovery, and 4) estimated CoM (eCoM) path displacement was calculated as the Euclidian distance in cm that the eCoM displaced from step initiation to first step recovery. The eCoM position in each frame was estimated as the mid-point between both anterior superior iliac spine markers. It was found earlier that during gait, the pelvis position is a reasonable proxy for the CoM position [15]. When a participant took extra steps to recover his/her balance, i.e., a multiple-step reaction, the following parameters were determined: 5) total balance recovery duration (ms) – the time from surface translation to foot contact on the ground, completing their multiple steps to recover balance; 6) recovery step path length – the Euclidian distance in cm that the ankle markers displaced from step initiation to foot contact on the ground, completing their full balance recovery; and 7) total eCoM path displacement (cm), defined as the distance in cm of the CoM traveled from the initial point prior to stepping to the point where participants completed their full balance recovery. Using this method, Batcir et al. [22] found excellent inter-observer reliability for first-step recovery initiation duration, step duration, step length, and the estimated distance of the eCoM traveled (ICC2,1 = 0.917, ICC2,1 = 0.975, ICC2,1 = 0.978, and ICC2,1 = 0.918, respectively; *p* < 0.001) and also for total balance recovery duration (ICC2,1 = 0.950, *p* < 0.001) and total recovery step path length (ICC2,1 = 0.935, *p* < 0.001) [22].

### Questionnaires and fall history

Height, weight, BMI, number of medications taken per day, number of diagnosed diseases, gender proportion, and data on fall histories were collected retrospectively via a face-to-face interview questionnaire. For fall prevalence, two questions were asked: 1) Have you fallen in the past 12 months? (“no” for 0 events, “yes” for 1 or more falls), and if they answered “yes,” they were asked: 2) How many times have you fallen in the past 12 months? A fall was defined as “an event that resulted in a person coming to rest inadvertently on the ground or other lower level regardless of whether an injury was sustained, and not as a result of a major intrinsic event or overwhelming hazard” [24]. We also assessed Mini Mental State Examination (MMSE) a 30-point questionnaire that measure cognitive impairment [25]. MMSE lower than 24 represent mild cognitive function. The “concerns about falling” was assessed by the Falls Efficacy Scale-International (FES-I) which is a reliable measure of fear of fall [26]. The evaluation of function was made using the Late Life Function and Disability Instrument (LLFDI) [27]. The functional component of the instrument contains 32 items that represent functional limitations, and inability to perform basic and advanced physical tasks in lower and upper extremities encountered in daily routines. Higher Late Life Function and Disability Instrument score indicates higher levels of balance function.

### Statistical analysis

All data were analyzed with PASW Statistics, version 23.0 (Somers, NY, USA). Prior to analysis, tests for assumptions of normality (the Shapiro-Wilk statistic) were performed. Group characteristics (NF, OF, RF) were assessed using a one-way ANOVA with a least significant difference (LSD) post-hoc test (age, weight, height, BMI), Kruskal-Wallis H test with pairwise-comparisons post-hoc analyses (medications per day, diagnosed diseases, self-reported retrospective falls, MMSE score, FES scores, and Late-Life function score), and chi-square for gender.

To examine the first hypothesis, a one-way ANOVA to analyze the differences in single- and multiple-step threshold levels between NFs, OFs, and RFs, in the case of significant differences (*p* < 0.05), an additional LSD post-hoc test was performed. Chi- square and Fisher’s exact tests were performed for comparing the probability of stepping between the groups. To test the associations between single- and multiple-step threshold levels and fear of falls (FES-I), we performed tests using Spearman’s correlation coefficient.

To examine the second hypothesis, we compared the kinematics of the recovery-step in the single-step and multiple-step threshold trials between the three groups using a one-way ANOVA. In the case of significant differences (*p* < 0.05), an additional LSD post-hoc test was performed.

To test our third hypothesis, we explored and compared the step recovery strategies that were performed in the three groups at different perturbation magnitudes using mosaic plots. A mosaic plot is a graphical display of the cell frequencies of a contingency table in which the area of boxes of the plot are proportional to the cell frequencies of the contingency table. The widths of the boxes are proportional to the percentage of steps performed out of the total stepping reactions. The heights of the boxes are proportional to the percent of the strategies used to recover from balance loss at each perturbation level. In addition, comparisons of frequencies of step recovery strategies between NFs, OFs, and RFs were made using chi-square and Fisher’s exact tests, and a Z test for comparing pairwise proportions when needed.

To examine the fourth hypothesis, we compared the kinematic parameters in the single-step and multiple-step reactions along all perturbation magnitudes of the three groups. These analyses required repeated and nested kinematic parameters for individual participants. To help determine differences in kinematic parameters for all stepping responses, we used a linear mixed-effects model with “subjects” as a random effect, the 13-perturbation magnitudes (“condition”) and the first step strategies (ULSS, LLSS, COS and leg collisions) as within-subjects’ factors, and group (NF, OF, or RF) as a between-subjects factor. Ten linear mixed-effects models were performed, four models for the kinematic parameters of the single recovery-step trials (e.g., step initiation, step duration, step length and eCoM path displacement) and six for the kinematic parameters of the multiple-step recovery trials (i.e., first recovery step-initiation duration, step length, total balance recovery duration, recovery step path length, and total eCoM path displacement). The final models included “group” (NF, OF, and RF) as a between-subjects factor controlling for the 13 perturbation magnitude levels (“condition”) and the strategy of the first recovery step that was executed as within-subjects’ factors. The significance level was set to *p* < 0.05.

## Results

As Table 1 shows, NFs had a significantly lower FES score than OFs (19 vs. 21, *p* = 0.009), and significantly higher overall function, basic lower extremity function, and advanced lower extremity function compared with RFs (67 vs. 57, *p* = 0.005; 81 vs. 70, *p* = 0.004; and 60 vs. 50, *p* = 0.008, respectively).

**Table 1:** Characteristics of Non-Fallers, One-Time Fallers, and Recurrent Fallers

Characteristic	Non-Fallers (*N* = 51)	One-Time Fallers (*N* = 20)	Recurrent Fallers (*N* = 12)	***p*** -value
**Age (year)**	79.6 ± 5.1	78.0 ± 5.5	78.5 ± 4.7	0.325
**Female % (number)**^a^	62.2 (33)	85.0 (17)	83.3 (10)	0.095
**Mini-Mental State Examination**^a^	29 (25,30)	29 (26,30)	29 (28,30)	0.448
**Number drugs/day**^a^	4.0 (0,10)	3.5 (1,11)	4.0 (2,9)	0.703
**Number of diagnosed diseases**^a^	1.0 (0,8)	2.0 (0,8)	1.0 (1,4)	0.665
**Height (cm)**	160.1 ± 10.3	155.3 ± 5.2	163.3 ± 10.4	0.051
**Weight (kg)**	67.1 ± 13.3	63.8 ± 8.2	78.0 ± 14.7	NF- OF: 0.592
				NF- RF: 0.030
				OF- RF: 0.010
**BMI (kg/m**^**2**^**)**	26.3 ± 4.2	26.4 ± 3.2	29.0 ± 3.7	0.097
**Number of falls in last year**^a^	0 (0,0)	1 (1,1)	2.5 (2,4)	NF- OF: < 0.001
				NF- RF: < 0.001
				OF- RF: 0.040
**Fall efficacy scale International (FES-I)**^a^	19 (16,34)	21 (16,58)	21 (16,39)	NF- OF: 0.009
				NF- RF: 0.938
				OF- RF: 0.078
**Late Life Function**				
Overall function^a^	67 (50,88)	66 (45,100)	57 (54,77)	NF- OF: 0.179
				NF- RF: 0.005
				OF- RF: 0.117
Upper extremity function^a^	19 (16,34)	21 (16,58)	21 (16,39)	0.483
Basic lower extremity function^a^	81 (61,100)	81 (53,100)	70 (65,88)	NF- OF: 0.115
				NF- RF: 0.004
				OF- RF: 0.162
Advanced lower extremity function^a^	60 (20,54)	59 (19,55)	50 (23,52)	NF- OF: 0.213
				NF- RF: 0.008
				OF- RF: 0.154

Values are means ±1 SD, where a variable was normally distributed using a one-way ANOVA with an LSD post-hoc test (age, weight, height, BMI), and medians (Min, Max) where a variable was not normally distributed using the Kruskal-Wallis H test with post-hoc tests to test pairwise comparisons (medications per day, diagnosed diseases, self-reported retrospective falls, MMSE, FES-I and Late-Life function scores) and chi-square for gender. *Abbreviations: cm* centimeters, *kg* kilograms, *kg/m*^*2*^ kilograms per meter square. ^a^ where a variable was not normally distributed

A total of 1999 fix of support and change of support balance recovery trials were observed (1268 for NFs, 445 for OFs, and 286 for RFs). Eighty-three participants reached their own single-step threshold level, 65 reached their own multiple-step threshold level (i.e., 15 subjects who completed the protocol did not reach their multiple-step responses, one participant asked to stop the experiment before reaching this threshold, and with two participants, we encountered technical problems at the end of the study protocol; thus, no multiple-step threshold trials were observed). The group comparison of kinematics of multiple-step events were made for these 65 participants.

### Single-step and multiple-step threshold levels

**Fig. 1:**
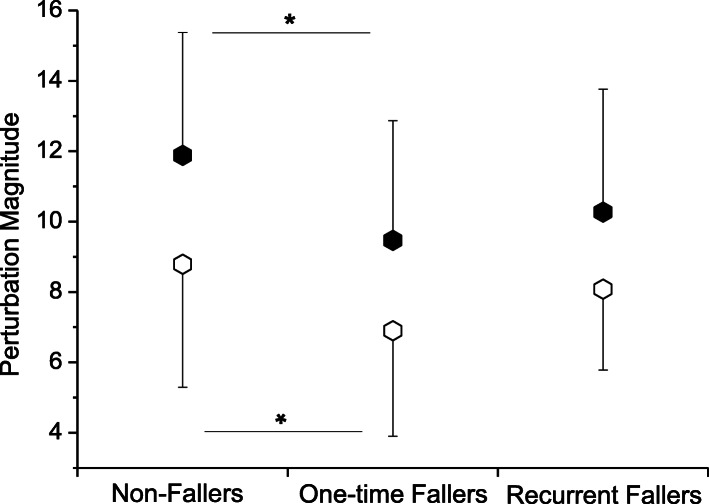
Single-step threshold (empty hexagon) and multiple-step threshold (filled hexagon) for NFs, OFs, and RFs. Placement of symbols indicates mean values; the whiskers of each plot indicate ±1 standard deviation. * Indicates significant differences between groups (*p* < 0.05) based on one-way ANOVA with LSD-post-hoc test

Figure 1 shows that compared with NFs, OFs had significantly lower single-step (8.90 ± 3.5 vs. 6.90 ± 3.0, *p* = 0.025) and multiple-step threshold levels (11.90 ± 3.5 vs. 9.47 ± 3.4, *p* = 0.009). There were no significant differences in these thresholds between NFs and RFs (8.90 ± 3.5 vs. 8.08 ± 2.3, *p* = 0.445 and 11.90 ± 3.5 vs. 10.27 ± 3.5, *p* = 0.141, respectively), as well as between OFs and RFs (6.90 ± 3.0 vs. 8.08 ± 2.3, *p* = 0.333 and 9.47 ± 3.4 vs. 10.27 ± 3.5, *p* = 0.552 respectively). Negative moderate associations were found between single-step threshold, multiple-step threshold, and FES-I (r = − 0.398, *p* < 0.001, and r = − 0.302, *p* = 0.007, respectively).

### Probability of stepping

**Fig. 2:**
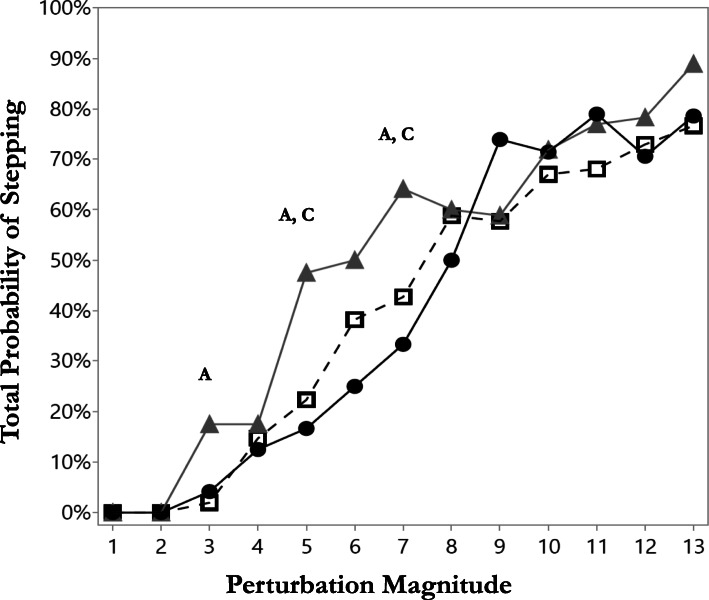
Mean probabilities of stepping by perturbation magnitude for non-fallers (NFs), one-time fallers (OFs), and recurrent fallers (RFs). Placement of symbols indicates mean values of the percentages of stepping trials out of the total number of trials at each perturbation magnitude for each group. The Fisher’s exact test (observed cases less than five) and chi-squared test (five or more observed cases) were used to test for differences in probabilities of stepping between groups. **a** – Significant differences between NFs and OFs. **b** – Significant differences between NFs and RFs. C- Significant differences between of OFs and RFs. Significance adjusted for 3-pairwise comparisons with a Bonferroni adjustment for multiple comparisons (*p* = 0.05/3 = 0.016)

Of 1999 trials, a total of 789 change of support trials were observed (NFs = 489, OFs = 196, and RFs = 104). The probability of stepping (single + multiple step trials) at low perturbation magnitudes were higher for OFs compared with NFs and RFs (Fig. 2) and reached significant differences at magnitudes 5 and 7 (47.5% vs. 22.3 and 16.7%, *p* = 0.004 and 64.1% vs. 42.7 and 33.3%, *p* = 0.008, respectively). Interestingly, the RFs exhibited the lowest probability of stepping until perturbation magnitude 7, then they exhibited a sharp increase in the probability of stepping responses at perturbation magnitude 8 or more.

### Kinematics of recovery stepping response at the single-step and multiple-step threshold trial

Table 2A shows that during the single-step threshold trials, RFs exhibited significantly longer step initiation duration, longer step duration, and larger eCoM displacement compared with NFs (*p* = 0.001, *p* = 0.001, and *p* = 0.002, respectively) and OFs (*p* = 0.003, *p* = 0.033, and *p* = 0.003, respectively).

**Table 2:** Recovery Step Kinematics for Non-Fallers, One-Time Fallers, and Recurrent-Fallers in (A) the Single-Step Threshold Trial. Note: 83 participants who reached their own single-step threshold levels were analyzed; and (B) the Multiple-Step Threshold Trial. Note: 65 participants who reached their multiple-step threshold levels were analyzed (see text for elaboration)

A. Single-Step threshold trial		**Non-Fallers (N = 51)**	**One-Time Fallers (N = 20)**	**Recurrent Fallers (N = 12)**	***p*-value**
***First recovery Step***	Step initiation duration (ms)	337 ± 88	340 ± 80	441 ± 95	NF- OF: 0.992
					**NF- RF: 0.001**
					**OF- RF: 0.003**
	Step duration (ms)	637 ± 99	695 ± 82	784 ± 157	NF- OF: 0.059
					**NF- RF: 0.001**
					**OF- RF: 0.033**
	Step length (cm)	5.8 ± 3.0	7.0 ± 3.4	9.6 ± 11.1	NF- RF: 0.091
	eCoM path displacement (cm)	5.8 ± 1.9	5.5 ± 2.1	8.1 ± 2.1	NF- OF: 0.678
					**NF- RF: 0.002**
					**OF- RF: 0.003**
B. Multiple-Step Threshold Trial		**Non-Fallers (*N*** **= 38)**	**One-Time Fallers (*N*** **= 17)**	**Recurrent Fallers (N = 10)**	***p*-value**
***Kinematic of Total balance recovery***	Recovery duration (ms)	1196 ± 246	1261 ± 394	1519 ± 324	NF- OF: 0.483
					**NF- RF: 0.009**
					OF- RF: 0.056
	Recovery step path length (cm)	32.5 ± 22.2	36.4 ± 26.3	44.6 ± 9.6	0.341
	Total eCoM path displacement (cm)	15.3 ± 9.7	14.9 ± 9.0	25.7 ± 11.4	NF- OF: 0.906
					**NF- RF: 0.007**
					**OF- RF: 0.012**
	Number of steps in the Multiple-Step threshold trial	2.31 ± 0.1	2.35 ± 0.1	2.7 ± 0.2	0.198

Values are mean ± 1SD. Linear mixed-effects models comparisons between the three groups based on a one-way ANOVA with a post-hoc (LSD) analysis for multiple comparisons. Significance level was set to *p* < 0.05. Abbreviations: *cm* centimeters, *ms* milliseconds

Table 2B shows that the first recovery-step initiation duration of the multiple-step threshold was not different between the groups. However, the total balance recovery duration was significantly longer, and the total eCoM displacement was significantly larger in RFs compared with NFs (1519 ms vs. 1196 ms, *p* = 0.009, and 25.7 cm vs. 15.3 cm, *p* = 0.007, respectively), indicating that RFs needed more time to fully recover from lateral balance loss. There were no statistically significant differences in the kinematics of stepping between NFs and OFs in the single-step or multiple-step threshold trials (Table 2).

### Stepping strategies at increasing magnitudes of perturbation

**Fig. 3:**
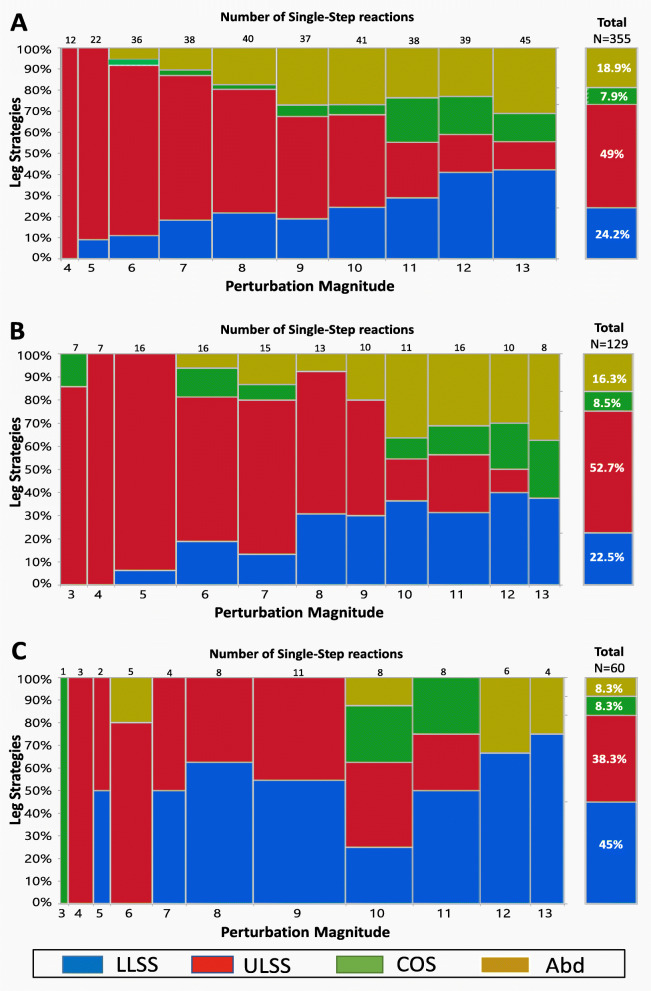
A mosaic plot showing the frequencies of the first recovery step strategies during single-step trials at increasing magnitudes of perturbation for non-fallers (**a**), one-time fallers (**b**), and recurrent fallers (**c**). Note: A mosaic plot is a graphical display of the leg strategy frequencies (Y-axis) by perturbation magnitudes (X-axis) during the single-step reactions. The widths of the boxes are proportional to the percentage of steps performed out of the total stepping reactions (the number of single-step reactions at each magnitude is presented at the top of each graph). The heights of the boxes are proportional to the percent of the strategies used to recover from balance loss at each perturbation magnitude level. The isolated right column summarizes all the frequencies of the leg strategies during all magnitudes. Abbreviations: LLSS, loaded leg side step; ULSS – unloaded leg side step; COS – crossover step; Leg Abduction – abducting the unloaded leg; Col - leg collisions

Mosaic plots in Fig. 3 A–C shows the recovery-step strategy frequencies during the single-step reactions. The total frequencies of the first recovery step strategies (Fig. 3 A–C, right columns) shows that the LLSS strategy accounted for 45% of RF recovery-step strategies, about two times more than OFs and NFs (22.5%, *p* < 0.001 and 24.2%, *p* < 0.001, respectively). Five percent of the recovery strategies of RFs during the single-step trials were unloaded leg strategies (38.3% ULSS, 8.3% COS and 8.3% leg-abduction), compared with 75.8% unloaded leg strategies in NFs (ULSS = 49%, COS = 7.8%, and Leg Abduction = 18.8%) (*p* < 0.001) and 77.5% in OFs (ULSS = 52.7%, COS = 8.5%, and Leg Abduction = 16.3%) (*p* < 0.001). Leg Abductions were seen rarely in RFs compared with NFs (*p* = 0.015) and OFs (*p* = 0.027). During the single-step trials, we observed a general decrease in ULSS strategies as the magnitudes of perturbation increased, especially in NFs and OFs, but RFs also tended to reduce the ULLS (Fig. 3 A–C).

**Fig. 4:**
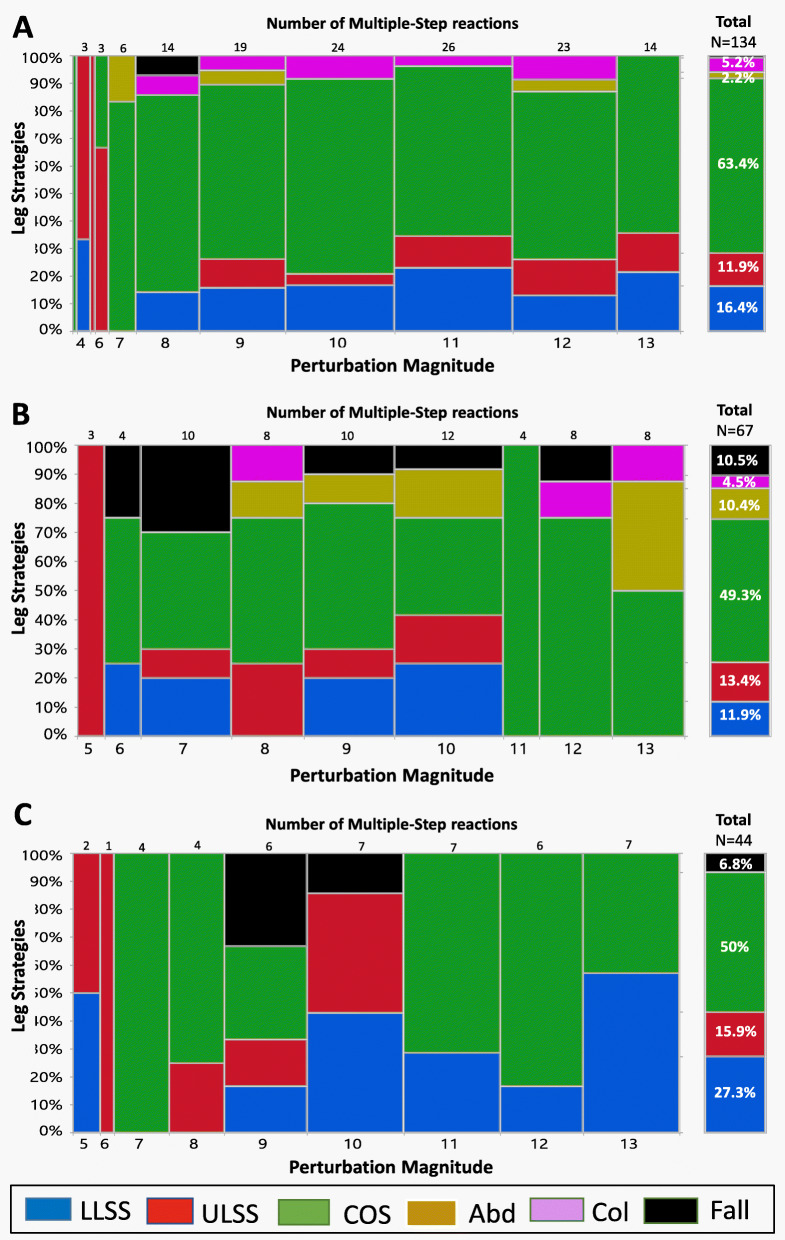
A mosaic plot showing the frequencies of the first recovery step strategies during multiple-step reaction trials at increasing magnitudes of perturbation for non-fallers (**a**), one-time fallers (**b**), and recurrent fallers (**c**). Note: A mosaic plot is a graphical display of the leg strategy frequencies (Y axis) by perturbation magnitudes (X axis) during the multiple-step reactions. The widths of the boxes are proportional to the percentage of steps performed out of the total stepping reactions (the number of multiple-step reactions at each magnitude presented at the top of each graph). The heights of the boxes are proportional to the percent of the strategies used to recover from balance loss at each perturbation magnitude level. The isolated right column summarizes all the frequencies of the leg strategies during all magnitudes. Abbreviations: LLSS, loaded leg side step; ULSS – unloaded leg side step; COS – crossover step; Leg Abduction – abducting the unloaded leg; Col - leg collisions

During the multiple-step trials, all three groups demonstrated the COS strategy as the most common step recovery strategy across all perturbation magnitudes, especially NFs (Fig. 4 A–C). Interestingly, during the multiple-stepping trials, 27.3% of the first step recovery strategies used by RFs were the loaded-leg strategy about two times more than OFs and NFs (11.9%, *p* = 0.043 and 16.4%, *p* = 0.097, respectively). The frequency of the unloaded leg strategies was 65.9% in RFs (ULSS = 15.9%, COS = 50%) compared with 82.7% in NFs (ULSS = 11.9%, COS = 63.4%, Col = 5.2% and Leg Abduction = 2.2%, *p* = 0.018), and 77.6% in OFs (ULSS = 13.4%, COS = 49.3%, Col = 4.4% and Leg Abduction = 10.4%, *p* = 0.176), with COS as the most common step recovery strategy in all three groups. Eleven falling events were observed in 0.9% of the multiple-step trials in NFs, compared with 6.8% for RFs (*p* = 0.007) and 10.5% for OFs (*p* < 0.001).

Additionally, we compared the leg strategy frequencies that were performed in the single-step reactions (Fig. 3 A–C) versus multiple-step reactions (Fig. 4 A–C). All three groups showed a decrease in performance of the “unloaded leg side step responses” (ULSS) and “loaded leg side step responses” (LLSS), with a dominance in performance of the COS strategy (X^*2*^ = 279.24, *p* < 0.001).

### Kinematics of recovery stepping response at increasing magnitudes of perturbation

Of a total of 789 change of support trials, 675 stepping reactions trials were included in the kinematic analysis (451 single-step and 224 multiple-step reactions). We excluded 114 stepping reactions from the analysis (11 fall trials and 103 leg abduction reactions). Ten linear mixed-effects models were performed: four for the single recovery-step reaction trials (e.g., step initiation, step duration, step length, and eCoM path displacement) and six for the multiple-step recovery reaction trials (i.e., first recovery step-initiation duration, step length, total balance recovery duration, recovery step path length, and total eCoM path displacement). Regarding the single-step reaction trials (see Table 3A for post-hoc tests), the model for step initiation duration (F = 9.72, *p* < 0.001) showed main effects for “group” (F = 5.26, *p* = 0.005) and for “first step strategy” (F = 14.63, *p* < 0.001), but not for perturbation magnitudes, i.e., “condition” (F = 0.14, *p* = 0.706), whereas the estimated means of RFs were slower compared with NFs and OFs. The models for recovery step duration (F = 23.97, *p* < 0.001) and eCoM path displacement (F = 156.03, *p* < 0.001) revealed main effects for “group” (F = 6.55, *p* = 0.002 and F = 5.29, *p* = 0.002, respectively), perturbation magnitudes, i.e., “condition” (F = 26.05, *p* < 0.001 and F = 144.33, *p* < 0.001, respectively) and “first step strategy” (F = 24.31, *p* < 0.001 and F = 88.10, *p* < 0.001, respectively). In regard to the step-length model (F = 59.35, *p* < 0.001), the main effects were found for perturbation magnitudes, i.e., “condition” (F = 34.06, *p* < 0.001) and “first step strategy” (F = 44.62, *p* < 0.001), with a trend toward significance noted for “group” (F = 2.45, *p* = 0.087).

**Table 3:** Post-hoc Analysis with Least-Significant-Difference Correction for Kinematic Parameter Comparison of Single-Step and Multiple-Step Reactions Controlled for Perturbation Magnitudes and First Step Strategies (Unloaded Leg Side Step, Loaded Leg Side Step, Crossover Step, Collision) of Non-Fallers, One-Time Fallers, and Recurrent Fallers

A. Single-Step Reactions	**Non-Fallers (*n*** **= 288)**	**One-time Fallers (*n*** **= 108)**	**Recurrent Fallers (*n*** **= 55)**	**Post-Hoc Test**		
	**Estimated mean ± SE (95% CI)**	**Estimated mean ± SE (95% CI)**	**Estimated mean ± SE (95% CI)**	**Comparisons**	**t**	**Adj*. p*-value**
Initiation duration (ms)	350 ± 8 (332–367)	344 ± 13 (318–370)	408 ± 17 (373–443)	NF vs. OF	−0.38	0.702
				**NF vs. RF**	−**3.06**	**0.002**
				**OF vs. RF**	−**2.96**	**0.003**
Step duration (ms)	674 ± 11 (651–698)	710 ± 18 (674–745)	765 ± 24 (717–812)	NF vs. OF	−1.73	0.084
				**NF vs. RF**	−**3.47**	**0.001**
				OF vs. RF	−1.85	0.064
Step length (cm)	14.04 ± 0.96 (12.16–15.93)	15.43 ± 1.44 (12.58–18.27)	18.60 ± 1.94 (11.48–19.69)	NF vs. OF	−0.84	0.402
				NF vs. RF	−2.18	0.030
				OF vs. RF	−1.33	0.182
eCoM path displacement (cm)	9.41 ± 0.39 (8.63–10.19)	10.72 ± 0.60 (9.54–11.91)	11.99 ± 0.80 (10.41–13.58)	NF vs. OF	−1.90	0.057
				**NF vs. RF**	−**2.98**	**0.003**
				OF vs. RF	−1.28	0.201
B. Multiple-Step ReactionsA.	**Non-Fallers (*n*** **= 130)**	**One-time Fallers (*n*** **= 53)**	**Recurrent Fallers (*n*** **= 41)**	**Post Hoc Test**		
	**Estimated mean ± SE (95% CI)**	**Estimated mean ± SE (95% CI)**	**Estimated mean ± SE (95% CI)**	**Comparisons**	**t**	**Adj*. p*-value**
***Total balance recovery***						
Recovery duration (ms)	1229 ± 66 (1098–1359)	1248 ± 96 (1058–1438)	1561 ± 115 (1333–1789)	NF vs. OF	−0.17	0.858
				**NF vs. RF**	−**2.64**	**0.009**
				OF vs. RF	− 2.15	0.032
Recovery step path length (cm)	31.01 ± 2.71 (25.65–36.37)	37.74 ± 3.99 (29.85–45.62)	40.46 ± 4.89 (30.80–50.11)	NF vs. OF	−1.46	0.145
				NF vs. RF	−1.77	0.078
				OF vs. RF	−0.44	0.658
Total eCoM path displacement (cm)	14.80 ± 1.38 (12.08–17.53)	15.93 ± 2.01 (11.94–19.91)	23.53 ± 2.48 (18.63–28.43)	NF vs. OF	−0.48	0.628
				**NF vs. RF**	−**3.24**	**0.001**
				**OF vs. RF**	−**2.45**	**0.015**

Significance level was set to *p* < 0.05/4 = 0.0125 for the single-step trials and *p* < 0.05/3 = 0.0167 for the multiple-step trials. n = number of step reaction trials analyzed

*Abbreviations: cm* centimeters, *ms* milliseconds, *SE* standard error, *CI* confidence interval

In the evaluation of the first recovery step of the multiple-step trials (Table 2B), the model of first recovery-step initiation duration (F = 2.75, *p* = 0.013) revealed a main effect for perturbation magnitudes, i.e., “condition” (F = 4.91, *p* = 0.028), and a trend was noted for “group” (F = 2.83, *p* = 0.061), but not for first stepping strategy. The model of the first step duration (F = 3.03, *p* = 0.007) showed a main effect for “first step strategy” (F = 3.70, *p* = 0.013) and a trend for “group” (F = 2.89, *p* = 0.057), but not for perturbation magnitudes, i.e., “condition”. The first-step-length model (F = 6.94, *p* < 0.001) revealed main effects for perturbation magnitudes, i.e., “condition” (F = 10.86, *p* < 0.001) and “first step strategy” (F = 6.31, *p* < 0.001), but not for “group” (F = 1.72, *p* = 0.181). Regarding the total balance recovery, the model of the total recovery duration (F = 2.10, *p* = 0.054) revealed a main effect for “group” (F = 3.61, *p* = 0.029), but not for perturbation magnitudes i.e., “condition” nor “first step strategy”. The recovery-step path-length model (F = 8.63, *p* < 0.001) showed main effects for perturbation magnitudes, i.e., “condition” (F = 24.10, *p* < 0.001) and “first step strategy” (F = 6.31, *p* < 0.001), but not for “group”. The model of the total eCoM path displacement (F = 5.94, *p* < 0.001) revealed main effects for “group” (F = 5.32, *p* = 0.006) and perturbation magnitude “condition” (F = 16.33, *p* < 0.001), but not for the first step strategy.

Regarding the step strategies in the single-step trials, post-hoc analyses revealed a significantly longer step initiation duration for the LLSS strategy compared with the ULSS and COS strategies. Step recovery time was longer during the COS strategy trials compared with the ULSS and LLSS strategies. During the COS trials, the step length was larger than in the LLSS step trials, while the step length in the LLSS was larger than in the ULSS. The eCoM path displacement during the LLSS was larger than in the COS, and the eCoM path displacement in COS was larger than in the ULSS.

The first step strategy of the multiple-step reactions revealed a significant difference for the step duration, whereas in the COS, the step duration was longer than in the LLSS, and the LLSS was longer than in the ULSS. Step length of both the LLSS and COS strategies were larger than in the ULSS. The total balance recovery step path length was longer in the COS strategy compared with both the ULSS and LLSS strategies.

## Discussion

As hypothesized, the levels of single-step and multiple-step thresholds were significantly lower in OFs compared to NFs (Fig. 1). Lower single-step and multiple-step thresholds were found earlier in older adults compared with young’s [22] and in stroke patients compared with age matched healthy controls [28]. A recovery step at a lower perturbation magnitude in OFs may suggest a lower balance recovery function, i.e., less ability to control the CoM motion over the BoS, or may be due to a past single fall event which resulted in a greater fear of falling (FES-I) that does not actually represent true balance abilities. A lower FES-I between OFs and NFs, a similar self-reported lower-extremity function between OFs and NFs (Table 1), as well as similar step recovery kinematics and step-recovery strategies (Tables 2 and 3), support the interpretation that due to a past single fall event, OFs have a more “responsive” over-reactive balance control system than NFs, and this does not truly represent their balance control abilities. Psychological factors related to postural threat are modulating cortical activity associated with postural reactions to unpredictable perturbations [29], it was found earlier that postural threat alters spinal reflex excitability reflected by soleus H-reflex attenuation [30]. This finding suggests that when postural control is threatened in older adults that have higher fear of fall such as in OFs, there is a shift to a more cortical involvement and a more responsive reaction.

Surprisingly, and in contrast to our hypothesis, the RFs single-step and multiple-step threshold levels were similar to that of NFs (Fig. 1). The question is why these thresholds in RFs were remarkably similar. First, the RFs in our study showed a low probability of stepping, especially at low perturbation magnitudes, suggesting less willingness to use the change BoS strategies, i.e., rapid recovery stepping responses at low perturbations, i.e., 1–8 (Fig. 2). Recovery stepping responses require rapidly lifting of the leg off the ground and then swinging it to the target location. This is also dependent on neuromotor mechanisms related to the build-up of muscle force and power to move the leg. Since RFs also reported lower low-extremity function (Table 1), it seems that they tried to avoid the use of a recovery step as much as they could. But when a recovery step was unavoidable at higher perturbation magnitudes, RFs in our study exhibited a delayed recovery step initiation duration in their single-step threshold trial compared with NFs (of about 100 ms), i.e., the lowest perturbation magnitude to evoke a recovery stepping response (Table 2A). This is further supported by the linear mixed-effects models of the single-step trials of all perturbation magnitudes that revealed that the step initiation duration of RFs was delayed compared with NFs and OFs (Table 3A). This, accompanied by larger recovery step length and a slower recovery step duration in RFs, compares with NFs and OFs (Tables 2A and 3A). In regard to the step recovery strategies, our findings show that the RFs used a more significant LLSS strategy compared with OFs and NFs (Fig. 3). It should be noted that in the LLSS strategy, there is a need to first unload the loaded leg, and then to swing the loaded leg sideways. Thus, we found that it took longer for RFs to lift the leg and initiate stepping (about 100 ms). Maki and Mcllroy [31] found that when the LLSS strategy was performed, the step initiation duration was delayed about 200 ms compared with the unloaded leg strategies (i.e., ULSS, COS, Leg Abduction). However, they found that when the LLSS strategy was used, the swing phase duration was shorter and step length was smaller [31]. In contrast, we found that the step length tended to be longer in RFs compared with both NFs and OFs. We assume that this resulted from the weaker ability of RFs to control the moving CoM over the BoS, i.e., a larger CoM displacement, resulting in a longer recovery step in the first recovery stepping (Tables 2A and 3A). The differences in recovery step length between Maki and Mcllroy [31] and our findings were due to a different population that was studied, i.e., younger people in Maki and Mcllroy’s study [31] versus older adults with a varying fall history in our study. Also, in Maki and Mcllroy [31], the participants were instructed to “try not to step”; thus, their younger participants tried to avoid stepping by decelerating the moving CoM over the BoS (a 200 ms delay in step initiation), and their recovery step length was shorter. We however, instructed our older adults to react naturally; thus, the larger first recovery step length was due to their lesser ability to decelerate the CoM, and a longer first recovery step was needed in RFs to catch the moving CoM.

Interestingly, the COS strategy was rarely seen at a low perturbation magnitude. At higher perturbation magnitudes, however, all three groups predominantly used the unloaded leg strategies, especially the COS strategy, which accounted for 49.3–63.4% of their balance strategies (Fig. 3). This was accompanied by a faster step initiation duration in the RFs in the multiple-step threshold trial versus their single-step trials (see Table 2A vs. B and Table 3A vs. B). The COS strategy was performed due the fact that higher perturbations magnitudes induced a faster CoM displacement to load the standing leg and unload the swing leg, allowing a more rapid foot-lift of the unloaded leg to “crossover” the loaded leg. The drawback of this strategy is that this was accompanied with a longer step recovery time (Table 2A vs. Table 2B), probably due to a more complex swing path to move the foot across the standing loaded leg (either in front of or behind it) with a larger eCoM path displacement in the first recovery step (Table 3A), and longer step recovery duration.

In regard to the multiple-step threshold trials, RFs had a somewhat longer first recovery step length than NFs (*p* = 0.066, Table 2B and *p* = 0.067, Table 3B). Although RFs had a longer first recovery step in the multiple-step trials, they were still unable to catch the moving CoM over the BoS, and the total eCoM path displacement was significantly larger (Table 2B and Fig. 3), resulting in a longer time to fully recover their balance (Tables 2B and 3B). Fujimoto et al. [32] also found that older fallers took multiple steps more often compared with older non-fallers, suggesting that this results from a reduced functional stability limit. They suggested that RFs have a pre-planned balance behavior in response to perturbation magnitudes that elicit a stepping response in which they take several small steps to recover balance instead of a one large single step that requires lower limb muscle power generation. Luchies et al. [33], Maki et al. [34], and Fujimoto et al. [35] suggested that during the execution of the first recovery step, the central nervous system (CNS) is already estimating the level of instability in foot contact and selects a more conservative protective stepping response. Luchies et al. [33] suggested that the CNS estimates the level of instability and preplans the correct balance recovery response and the use of extra small steps to recover balance even before the first recovery step is completed. This suggests that to ensure full recovery immediately after the first recovery step response, the CNS is estimating the level of instability and selects a recovery stepping response based on the subject’s past fall experience and physical abilities. It was previously reported by Maki and Mcllroy [31] that when subjects performed repeated trials, the prevalence of multiple steps increased. This further suggests a preplanning motor function. Our results show that due to the inability to control the CoM path displacement, the RFs were more likely to take extra steps in order to avoid falls and to regain more opportunities to make corrective adjustments in their response following a lateral loss of balance (Tables 2B and 3B). Work by Pai and Patton [36] and Pai et al. [37] demonstrated that the occurrence of a step depends on the interaction between the CoM position and its velocity. Following their model, stepping is necessary if there is a sufficiently high velocity of CoM displacement, even if the vertical projection of the CoM is located within the BoS at step initiation, i.e., a concept based on CoM velocity-position limits.

The main limitation of the present study was use of a convenience sample of relatively healthy community dwelling older adult volunteers and of retrospective fall reports. A prospective study that monitors falls prospectively and includes older adults with a different functional status (i.e., healthy, pre-frail, and frail older adults) might resolve this issue. A second limitation is that this sample of RFs was small and limits the interpretation of the results. Thus, it might have been too small to reveal a significant differences in all kinematic parameters of recovery stepping. A third limitation is that the observation of arm and trunk motion was not reported in this report. It was previously reported that these movements helped to preserve balance and decelerate CoM motion during loss of balance. Further analysis might help to identify how these recovery arm and trunk movements are associated to a systematically increase of perturbation magnitudes. A fourth limitation is that the laboratory perturbations in the present study that were based on surface translation during standing may not mimic a real-life situation of balance loss closely enough due to pushes and trips.

## Conclusion

This study showed that while older adults who experienced a coincidental single fall event (OFs) showed a lower step threshold, a more “responsive” over-reactive step response related from their fear of falling and not due to impaired balance abilities. The poorer balance recovery function in RF were associated with poorer self-reported lower extremity function and less able to rapidly lift the stepping leg off the ground thus, they tried to avoid stepping and use fix of support strategy to restore balance. When step response was inevitable the LLSS first step strategy was performed by RFs at a low perturbation magnitude, which slowed down step initiation. This resulted in an increase in the CoM displacement, causing longer step duration to complete the stepping vs. NFs. At higher perturbation magnitudes, when multiple steps were needed to preserve balance and prevent falling, all three groups used the unloaded leg strategies (i.e., CoS or ULSS) as their main balance recovery strategies. The use of these strategy resulted a faster step initiation (i.e., faster leg lift-off) however, RFs still showed a tendency to a longer first step length compared with NFs, causing a larger CoM displacement; thus, more time to fully recover from unexpected balance loss (Tables 2B and 3B) suggesting impaired ability to control of the CoM motion over BoS. Our results suggests that the differences in step kinematic and CoM characteristics does not reflect the different types of strategies taken. Rather, it reflects direct impairments in performance within a given step type in RFs. Thus, for older RFs specific balance recovery training to improve the skill of rapidly controlling the CoM motion is required.

## Supplementary information

**Additional file 1: Table S1.** Characteristics of Surface Horizontal Translation.

## Data Availability

Data will be available upon request.

## Abbreviations

NFs: non-fallers
OFs: one-time fallers
RFs: recurrent-fallers
FES-I: Falls Efficacy Scale-International
CoM: center of mass
eCoM: estimated CoM
BoS: base of support
LLSS: loaded leg side step
ULSS: unloaded leg side step
COS: crossover step
Col: leg collision
ms: milliseconds
cm: centimeters
BMI: Body Mass Index
LLFDI: Late-Life Function and Disability Instrument
LSD: least significant difference
