# Supplementary material for: The kinematics and strategies of recovery steps during lateral losses of balance in standing at different perturbation magnitudes in older adults with varying history of falls
Source: BMC Geriatr. 2020 Jul 20;20:249. doi: 10.1186/s12877-020-01650-4 (PMC7372810; doi:10.1186/s12877-020-01650-4)
Supplement: Supplementary file 1 — Additional file 1: Table S1. Characteristics of Surface Horizontal Translation. [file 12877_2020_1650_MOESM1_ESM.docx]

**Table 1 – Supplementary:** Characteristics of Surface Horizontal Translation

| **Displacement** (cm) | **Displacement Time** (sec) | **Velocity** (cm/sec) | **Acceleration** (cm/sec^2^) |
| --- | --- | --- | --- |
| **1** | 0.30 | 6 | 25 |
| **2** | 0.40 | 7 | 60 |
| **3** | 0.45 | 11 | 80 |
| **4** | 0.50 | 14 | 85 |
| **5** | 0.55 | 15 | 90 |
| **6** | 0.60 | 17 | 94 |
| **7** | 0.65 | 19 | 98 |
| **8** | 0.68 | 21 | 102 |
| **9** | 0.70 | 22 | 107 |
| **10** | 0.73 | 23 | 111 |
| **11** | 0.75 | 25 | 117 |
| **12** | 0.78 | 26 | 123 |
| **13** | 0.80 | 28 | 128 |
| **14** | 0.83 | 30 | 133 |
| **15** | 0.85 | 31 | 139 |
| **16** | 0.86 | 33 | 146 |
| **17** | 0.87 | 34 | 151 |
| **18** | 0.88 | 36 | 158 |

The basic protocol on which all analyses were carried out included 13 perturbation magnitudes ranging from 1 to 13 (a total of 26 random right and left perturbation trials, Supplementary Table 1) until the participants reached their stepping thresholds. In case participants did not reach their stepping thresholds during the basic protocol, after their agreement, they continued until reaching the stepping thresholds or until a perturbation magnitude of 18 to a maximum horizontal surface translation of 18cm. At any point, they were able to take a rest break or stop the experiment.

cm = centimeters; sec = seconds; sec/cm = centimeters per second; sec/cm^2^ = centimeters per second squared
